# Supplementary material for: Patterns of Intron Gain and Loss in Fungi
Source: PLoS Biol. 2004 Nov 30;2(12):e422. doi: 10.1371/journal.pbio.0020422 (PMC532390; doi:10.1371/journal.pbio.0020422)
Supplement: Table S1 — Also available at http://genes.mit.edu/NielsenEtAl/. (4.3 MB ZIP). [file pbio.0020422.st001.zip › NielsenEtAl/html/408.html]

AN8748.1.NCU02027.1.MG02811.1.FG06404.1


```
 CLUSTAL W (1.82) Multiple Sequence Alignments - Introns Inserted


Sequence 1: NCU02027.1	245 aa
Sequence 2: MG02811.1	271 aa
Sequence 3: FG06404.1	235 aa
Sequence 4: AN8748.1	239 aa
Alignment Length: 276 aa
Number Identitical Residues: 89 aa
Alignment Score (without introns) 4912


MG02811.1 	MPNYP--APLRFPAAGRHTATVIFIHGLGDS~GHGW~APAVENWRRRQKLDEVKFILPHA
NCU02027.1	MASLARRPPLLVPAVARHTATVIFIHGLGDT~GHGW~ASAVEQWRRRQRLDEVKFILPHA
FG06404.1 	MSSGR-IAPLVFPAASQHTATVIFVHGLGDT~GHGW~ASAVENWRRRQKMDEVKFILPHA
AN8748.1  	MSR----APFIVPALKKHTATVIMAHGLGDS2GAGW2VSLAHNWRRRGLFEEVTFIFPNA
          	*.     .*: .**  :******: *****: * ** .. ..:****  ::**.**:*:*

MG02811.1 	PTIPVTC~N0MGMRMPGWYDI0KSIDG-~-TPESLRKDEDEEGILLSQKYFHELIQQEID
NCU02027.1	PSIPITA~N~WGMKMPGWYDI0FAIDG-~-SAEALRRNEDEAGILTSQAYFHDLIQKEID
FG06404.1 	PQIPISV0N~MGMRMPGWFDI0KQLGG-~-DVDSLIRNEDTEGIKLSQKYFHNLIQQEID
AN8748.1  	PMIPITV0N~FGMSMPGWYDI~TKLGRD0LDFQEAVKNQDEAGILKSRDYFNSLIKEQMD
          	* **::  *  ** ****:**   :. .    :   :::*  **  *: **:.**::::*

MG02811.1 	AGIPSERIVLGGFSQGGVMSIFSGLTAKVKLAAIVAMSAYVPLSLKFKELVASCEANKAT
NCU02027.1	SGIPADRIVIGGFSQGGAMGLFSGLTAKCKLAGIIALSSYLLLSLKFAELVPKPEFNKET
FG06404.1 	SGIVPERIVLGGFSQGGAMSLLAGLTCTSKLGGILGLSSWLLLSKTFADMVKPTDANRQT
AN8748.1  	QGIKPSRIVLGGFSQGGAMSLFSGITGQEKLGGVFGLSCYMLLSDRIKNYIPENFPNKKT
          	 ** ..***:*******.*.:::*:*   **..:..:*.:: **  : : :     *: *

MG02811.1 	PIWMGHGTTDLVVPTVLGMMSEALLKDEGYQ-VSMKLYP2GMGHSACPEELDEVEAFLRK
NCU02027.1	PIFMAHGDADPVVNYKLGTMTRDLLKEMGYN-VKFTTYP2GMGHSACLEELDAIEDFLTE
FG06404.1 	PVMMFHGEEDPIVPCERGKLSAELLKGLGYD-VAWKTYP2GMGHSAVPEELDEVEAFLRK
AN8748.1  	PFFLAHGTEDDIVPHEFGKRSAEMAKELGLEDVTFNSYK2YLSHSADPVEIEDLEKFLDR
          	*. : **  * :*    *  :  : *  * :.*  . *   :.***   *:: :* ** .

MG02811.1 	SLPPLGNTGSGV1LHQSTSRNRPHIREEECDPGGKLAPRNLGKY
NCU02027.1	RLPKVADK----~-----SEQKSEL-------------------
FG06404.1 	QLPPKN------~-------------------------------
AN8748.1  	VIPAENGGSL--~-------------------------------
          	 :*   . .
```
